# Implicit and Explicit Self-Esteem in Current, Remitted, Recovered, and Comorbid Depression and Anxiety Disorders: The NESDA Study

**DOI:** 10.1371/journal.pone.0166116

**Published:** 2016-11-15

**Authors:** Lonneke A. van Tuijl, Klaske A. Glashouwer, Claudi L. H. Bockting, Jorge N. Tendeiro, Brenda W. J. H. Penninx, Peter J. de Jong

**Affiliations:** 1 Department of Clinical Psychology and Experimental Psychopathology, University of Groningen, Groningen, The Netherlands; 2 Department of Clinical Psychology and Experimental Psychopathology, Utrecht University, Utrecht, The Netherlands; 3 Department of Psychometrics and Statistics, University of Groningen, Groningen, The Netherlands; 4 Department of Psychiatry, VU University Medical Center, Amsterdam, Amsterdam, The Netherlands; Stellenbosch University, SOUTH AFRICA

## Abstract

**Background:**

Dual processing models of psychopathology emphasize the relevance of differentiating between deliberative self-evaluative processes (explicit self-esteem; ESE) and automatically-elicited affective self-associations (implicit self-esteem; ISE). It has been proposed that both low ESE and ISE would be involved in major depressive disorder (MDD) and anxiety disorders (AD). Further, it has been hypothesized that MDD and AD may result in a low ISE “scar” that may contribute to recurrence after remission. However, the available evidence provides no straightforward support for the relevance of low ISE in MDD/AD, and studies testing the relevance of discrepant SE even showed that especially high ISE combined with low ESE is predictive of the development of internalizing symptoms. However, these earlier findings have been limited by small sample sizes, poorly defined groups in terms of comorbidity and phase of the disorders, and by using inadequate indices of discrepant SE. Therefore, this study tested further the proposed role of ISE and discrepant SE in a large-scale study allowing for stricter differentiation between groups and phase of disorder.

**Method:**

In the context of the Netherlands Study of Depression and Anxiety (NESDA), we selected participants with current MDD (n = 60), AD (n = 111), and comorbid MDD/AD (n = 71), remitted MDD (n = 41), AD (n = 29), and comorbid MDD/AD (n = 14), recovered MDD (n = 136) and AD (n = 98), and never MDD or AD controls (n = 382). The Implicit Association Test was used to index ISE and the Rosenberg Self-Esteem Scale indexed ESE.

**Results:**

Controls reported higher ESE than all other groups, and current comorbid MDD/AD had lower ESE than all other clinical groups. ISE was only lower than controls in current comorbid AD/MDD. Discrepant self-esteem (difference between ISE and ESE) was not associated with disorder status once controlling for ESE.

**Limitations:**

Cross-sectional design limits causal inferences.

**Conclusion:**

Findings suggest a prominent role for ESE in MDD and AD, while in comorbid MDD/AD negative self-evaluations are also present at the implicit level. There was no evidence to support the view that AD and MDD would result in a low ISE “scar”.

## Introduction

Self-reported low self-esteem has consistently been observed in episodes of major depressive disorder(MDD; [1]) and in episodes of anxiety disorders (AD; [2]) like social anxiety disorder, panic disorder, and generalized anxiety disorder. It is unsurprising that low self-esteem is prevalent in MDD given that it is one of the possible symptoms as outlined in the most recent version of the Diagnostic and Statistical Manual of Mental Disorders (“feelings of worthlessness”; [3]). In a meta-analysis [4], low self-esteem was shown to predict prospective symptoms of depression and anxiety, highlighting a potential causal role in MDD and AD etiology. The authors argued that low self-esteem might lead to social disruptions, increased self-focus, and rumination, which would in turn lead to the development (and maintenance) of depressive symptoms. For symptoms of anxiety, high self-esteem has been argued to act as an anxiety buffer [5], and therefore low levels of self-esteem would make an individual more vulnerable to anxious thoughts and feelings. Indeed, those who had been manipulated with a self-esteem boost reported less anxiety following a traumatic video than those who had received a neutral self-esteem manipulation [5]. As such, the presence and relevance of low self-esteem in both AD and MDD is fairly indisputable. In the present article, we use the term “episode” also for the duration that a person meets the criteria for an AD without recovery.

Self-esteem has been predominantly indexed by self-report measures, which are limited by what the respondent is willing and able to disclose (e.g., [6,7]). Meanwhile, relevant self-associations may not necessarily be accessible for conscious introspection, and individuals may dismiss certain initial associations as irrelevant when asked to verbalize a global affective self-evaluation. This points to the importance of complementing self-report measures reflecting the “self-endorsed”, deliberate self-evaluations (explicit self-esteem; [8]) with measures of automatic associations that require neither verbalization nor introspection (implicit self-esteem; cf. [9]). The relevance of differentiating between explicit (ESE) and implicit self-esteem (ISE) is further emphasized by the view that both facets of self-esteem are differentially involved in more controlled/strategic versus more automatic/spontaneous behaviors. ESE is considered to be especially relevant in the context of more deliberative/reflective behavior (e.g., choosing not to speak up in a group discussion), while ISE is argued to be critically involved in more reflexive, spontaneous behaviors (e.g., blushing during public speaking; [10]). In support of this, Spalding and Hardin [11] found that those low in ISE, as indexed with an affective priming task, displayed more observer-rated anxious behaviors during an interview, specifically when the interview involved self-related questions. A more recent study found that ESE was related to self-reported measures of anxiety/nervousness, and controlled nonverbal behaviors during a speech (e.g., hand gestures to emphasize the point verbally made), while ISE, as indexed by both a cognitive load task and the implicit association test (IAT), was related to general observer-rated anxiety and spontaneous nonverbal behaviors during a speech (e.g., nervous mouth movements; [10]). Given the distinct roles of ISE and ESE in automatic and controlled dysfunctional (pathogenic) behaviors, it is feasible that these two components of self-esteem are (partly) differentially involved in AD and MDD. As measures of ISE are thought to capture different aspects of the construct [12], it is important to note which ISE measure has been used previously as this may explain inconsistencies in findings.

Empirical evidence for the presence of low ISE in AD and MDD is mixed. Studies looking at ISE in AD samples are relatively few and seem to have focused almost exclusively on social anxiety disorder (symptoms). Two analogue studies using an IAT as a measure of ISE found relatively low ISE in females scoring high on symptoms of social anxiety [13,14]. Consistent with this, a correlational study focusing on adolescents showed a negative relationship between symptoms of social anxiety and ISE measured with an IAT [15]. A more recent small-scale clinical study found that patients with social anxiety disorder (n = 45) had lower ISE, as indexed by an IAT, than healthy controls [16]. Patients with panic disorder (n = 24) showed a similar tendency that was not significant, possibly due to low statistical power. Concerning MDD, a meta-analysis of 25 studies (n = 2831, of which 77% non-clinical) indicated that there is an association between low ISE and symptoms of depression [17]. However, when focusing only on studies using clinical, adult depressed samples, many have failed to find lower ISE in comparison to those who have never been depressed [18–21]. Risch et al. [22] did, however, find lower ISE in current MDD patients (both first-onset and recurrent) compared to healthy controls, when using an IAT. Franck, De Raedt, Dereu and van den Abbeele [23] also found evidence for lowered ISE in current MDD patients using an IAT but only in those without suicidal ideation, while MDD patients with suicidal ideation did not differ from never-depressed controls. As such, despite the strong theoretical grounds for anticipating low ISE in MDD and AD, the evidence thus far, particularly for MDD, is not very convincing, and suggest that dysfunctional self-related thoughts and behaviors may be most pronounced at the deliberate, explicit level.

Previous studies involving clinical samples may simply not have had enough power to detect an effect of ISE. Further, as little is known about ISE in comorbid MDD and AD, and many studies looking at MDD do not have anxiety disorder diagnosis as an exclusion criterion (e.g., [21,22]), it is important to look at ISE not only in comorbidity, but in (relatively) pure forms of MDD and AD as well. This may elucidate the apparently inconsistent earlier findings regarding ISE in MDD and AD. Therefore, the present study was designed in the context of a large-scale, national study: the Netherlands Study of Depression and Anxiety (NESDA; www.nesda.nl/). Specifically, ISE (and ESE) in (relatively) pure forms of MDD and AD, as well as in individuals with comorbid MDD/AD were analyzed. Given previous reports of relatively high reliability [12] with strong evidence of validity [10], the IAT was used to measure ISE. In addition, we took suicidal ideation into account to test the robustness of the earlier finding that only in the absence of suicidal ideation is MDD associated with lowered ISE [23].

Given that low self-esteem is prevalent in both depression and anxiety, it seems feasible that self-esteem is a transdiagnostic factor which may explain the high rates of comorbidity between the two [24,25]. Indeed, low self-esteem may increase vulnerability for both depression and anxiety, and other factors may then determine, specifically, which of the two develops. Consistent with this view, a relatively large scale study among non-referred adolescents found that the association between symptoms of depression and social anxiety could largely be explained by adolescents’ explicit self-esteem [15]. This earlier study provided no evidence for a unique relationship between low self-esteem and the covariation of (social) anxiety and depressive symptoms, but rather independent relationships between low self-esteem and the severity of depression and anxiety symptoms. This suggests that low self-esteem may increase the chance of concurrent symptoms of anxiety and depression by independently increasing the chance of developing both types of symptoms. Given that this earlier study was limited to a non-clinical sample of adolescents, it remains to be seen whether self-esteem can (partly) explain the high rates of comorbidity in an adult sample containing many who meet the clinical criteria for a depression and/or anxiety disorder. Therefore, the relationship between depressive and anxious symptoms are analyzed in the present study, once controlling for self-esteem.

It is important to see to what extent low self-esteem will be normalized when remitted/recovered from a depressive episode or anxiety disorder. Given the high rate of recurrence that is typical of both MDD and AD, identifying possible “scars” that remain following episodes (e.g., lowered ISE) may be crucial in identifying those who will relapse [26]. Even when AD and MDD enters remission, or recovery, it is important to differentiate between ISE and ESE. It is feasible that those who are in remission/recovery are able to address negative self-related thoughts, and are motivated to reappraise a situation in order to derive at a more positive self-evaluation. Indeed, self-awareness and addressing negative thoughts effectively is the core aim of most therapies (e.g., cognitive behavioral therapy). However, given the lack of control over ISE, spontaneous (pathogenic) behaviors may continue that are not necessarily within the realm of awareness. Consistent with this, Vasey, Harbaugh, Buffington, Jones and Fazio [27] found that implicit associations, as measured with an IAT, following an exposure therapy predicted return of fear in public-speaking phobia. Despite this, two studies reported that ISE and ESE in remitted MDD did not differ from never-depressed controls, both of which had used the IAT [18,22]. Given the small sample sizes used, it is important to test the hypothesis in larger samples, and also take AD and comorbid MDD and AD into consideration as distinct disorders with distinct etiologies. As ISE may improve over time through consistent high ESE that becomes overlearned and the default reaction [28], it is also important to differentiate between those whose symptoms have recently remitted and those who no longer meet the criteria for MDD and AD for some time (recovered). The present study therefore included remitted and recovered MDD, AD and comorbid MDD/AD groups.

For some individuals, ESE and ISE may differ considerably (i.e., *discrepant self-esteem*; [29]). While *fragile self-esteem* refers to the pattern of low ISE and high ESE, and has been linked to narcissistic tendencies [30], *damaged self-esteem* refers to the pattern of high ISE and low ESE, and has been linked to symptoms of depression [31]. While some studies looked at discrepancy by including the interaction between ISE and ESE into the model [32], others argued that this fails to acknowledge the potential influence of the direction of the discrepancy [33]. That is, the extent that ISE and ESE differ may only be related to symptoms of depression or anxiety when, for example, ISE is higher than ESE. Without distinguishing the direction of discrepancy, the interaction may appear statistically non-significant. Other studies looking at self-esteem discrepancies adopted analyses that allowed for differentiating between the direction of the discrepancy (i.e., fragile or damaged), but did not allow for the inclusion of the main effects (i.e., ESE and ISE; [34]). The inclusion of ESE into the model resulted in an issue of multicollinearity. Given strong relationships between ESE and psychopathology are often reported, previously observed associations between damaged self-esteem and depression may have simply been an artefact of ESE, regardless of ISE. The final aim of the present study is therefore to explore an alternative method to analyse the role of discrepant self-esteem in MDD and AD which allows for differentiating between fragile and damaged self-esteem while statistically controlling for the potential main effect of ESE.

All in all, the present study tested the following hypotheses: i) those without a lifetime diagnosis of depression or anxiety (i.e., the comparison group) will show higher ESE and ISE than current MDD, AD, and comorbid MDD/AD; ii) ISE and ESE in the comparison group will also be higher than those who were recovered or remitted from AD, MDD, or comorbid MDD/AD; iii) those who have recovered or remitted from AD, MDD or comorbid both, will have higher ISE and ESE than those who currently meet the diagnosis; iv) self-esteem is a transdiagnostic factor, and as such, levels between MDD, AD and comorbid MDD/AD will not differ at the remitted level, the recovered level and at the current diagnosis level. Further, a novel way of testing the presence of self-esteem discrepancies in those with a current depression or current anxiety disorder is explored that allows for the inclusion of ESE as a main effect in the model.

## Materials and Methods

### Participants

The Netherlands Study of Depression and Anxiety (NESDA; www.nesda.nl/) is an ongoing longitudinal cohort study that, at baseline (2004–2007), involved 2981 participants who have been followed-up biannually across a number of measures. In order to follow the long-term course of depression and anxiety, 1701 participants with a current depressive or anxiety disorder, and 907 participants with a life-time diagnosis or at-risk (e.g., subthreshold symptoms), were recruited from the community, primary care, and mental health organisations. A further 373 participants with no current or history of any depressive disorder or AD were recruited as controls. There were two exclusion criteria in the NESDA: 1) A primary, clinically overt diagnosis of a psychotic disorder, an obsessive-compulsive disorder, a bipolar disorder, or a severe addiction; 2) Non-fluent command of the Dutch language. A thorough overview of the recruitment process, design, and overarching aims of NESDA are published elsewhere [35]. All participants provided written consent, and ethical approval was granted by all ethical committees of participating universities (VU University Medical Center, Leiden University Medical Center and University Medical Center Groningen).

The present study makes use of data collected in the most recent wave at the time of writing, which is approximately 6 years since baseline, and the fourth biannual measurement. At this wave, 2256 participants were measured (24% attrition since baseline), where 1799 (80%) received the measures relevant for the present analysis. The remaining 457 did not receive all the measures for various technical reasons (e.g., completing measures at home or over the phone prohibiting computer-based measures). Participants who were given all relevant measures were aged between 23 and 72 (M = 48.05, SD = 13.18; 63.6% female). Participants were excluded from the present analysis if they had developed a bipolar disorder at some point during the study, or reported an alcohol dependence since the last interview (approximately 2 years ago; n = 83).

Clinical groups were formed for MDD and AD, and split by those currently in an episode (diagnosis in past month), those in remission (an episode that had ended in the last six—one month), and those recovered (an episode in the last seven years–six months). We used these cut-offs as these were more readily available within the study. It should be noted that what defines, for example, a depression in remission varies across studies. Frank et al. [36] recommends that remission be considered as a depression-free period of 2–6 months, with longer than 6 months considered a recovery. Our cut-offs are not too far from this. Cut-offs for ADs are dependent on the type of AD, however we apply the same cut-offs as used for MDD for consistency when comparing the groups and creating comorbid groups. To create a more homogeneous group, participants were excluded from the current and remitted AD groups if they had also met the criteria for MDD (or dysthymia; n = 135 & 27, respectively) since the last interview. Likewise, those with an AD since the last interview were excluded from the current and remitted MDD groups (n = 78 & 45, respectively). Those in the recovered AD or MDD groups had no history of MDD (and dysthymia) or AD, respectively. Current and remitted comorbid AD and MDD (CM) groups were also formed based on the same criteria as the MDD and AD groups. Those who had comorbid dysthymia in the current CM and MDD groups were not excluded. A recovered CM group was not created given that the available information made it difficult to determine whether AD and MDD had occurred and ended at the same time. The comparison (control) group consisted of individuals without a history of AD, MDD or dysthymia. The upper half of Table 1 provides an overview of the demographics and size of each group.

**Table 1:** Means (& standard deviations; unless stated otherwise) of demographics and variables per group.

	Major Depressive Disorder (MDD)			Anxiety Disorder[s] (AD)			Comorbid MDD & AD		Comparison
	Current (n = 60)	Remitted (n = 41)	Recovered (n = 136)	Current (n = 111)	Remitted (n = 29)	Recovered (n = 98)	Current (n = 71)	Remitted (n = 14)	Non-Clinical (n = 382)
Age	49.05 (12.65)	49.02 (12.84)	46.95 (13.29)	48.85 (12.23)	45.45 (12.12)	47.56 (13.83)	46.90 (11.17)	44.93 (12.39)	48.23 (14.53)
Female (%)	68.3	70.7	61.8	70.3	75.9	57.1	69.0	71.4	57.1
BAI	12.85 (8.04)	9.38 (6.09)	5.47 (5.15)	14.03 (9.63)	11.17 (8.24)	6.16 (4.79)	20.32 (10.17)	9.08 (6.65)	2.74 (3.48)
IDS	28.05 (9.82)	19.83 (7.51)	12.26 (8.99)	20.74 (10.59)	16.14 (8.45)	11.42 (7.01)	33.86 (10.85)	18.17 (8.16)	5.46 (4.74)
Invalid IAT (n)	5	3	8	5	2	8	11	1	25
RSES	26.13 (5.24)^e^	27.71 (4.53)^ed^	32.45 (4.24)^c^	28.44 (5.11)^ed^	30.31 (5.23)^cd^	31.65 (4.57)^c^	23.07 (4.98)^b^	27.50 (3.88)^ed^	35.18 (3.98)^a^
IAT	.62 (.47)^ab^	.63 (.50)^ab^	.65 (.45)^a^	.61 (.44)^ab^	.48 (.56)^ab^	.64 (.47)^a^	.41 (.47)^b^	.54 (.41)^ab^	.74 (.43)^a^

*Note*. BAI = Beck Anxiety Inventory; IDS = Inventory of Depressive Symptomatology; RSES = Rosenberg Self-Esteem Scale; IAT = Implicit Association Test; Current = episode in the past month; Remitted = episode ended one–six months ago; Recovered = episode ended 6 months– 7 years ago.

For rows RSES and IAT: means with the same superscripts did not differ significantly (*p* > .05).

### Measures

#### Beck Anxiety Inventory (BAI; [37])

The BAI is a self-report questionnaire measuring the severity of 21 anxiety symptoms in the past week (e.g., “Nervous”, “Hot/cold sweats”). The degree of botheration is answered on a 4-point Likert scale from 1 (*Not at all*) to 4 (*Severely [I could barely stand it]*). Total scores were calculated (possible range: 21–84), with higher scores indicative of relatively more anxious symptoms in the preceding week. Missing answers were replaced with participant’s mean response (n = 47). From the 1799 participants, 29 participants failed to return the questionnaire and four had more than nine missing answers; these were excluded from any analysis involving the BAI. The BAI showed excellent internal reliability across all those without missing answers (n = 2084; Cronbach’s α = .92).

#### Inventory of Depressive Symptomatology–self-report (IDS; [38])

The self-report IDS was used to measure depressive symptomatology in the preceding seven days, based on the DSM-IV criteria for MDD. In the original version, at two points, participants can chose to answer one of two items (e.g., “Decreased appetite” or “Increased appetite”), and therefore answer 28 of the 30 items in total. The version in NESDA combines the paired items, and therefore contains 28 items, all of which are answered by the participant. For each of the 28 items (e.g., “Feeling sad”) there are four corresponding answers from “0” that is indicative of no depression (e.g., “I do not feel sad”) to “3” referring to a more severe depressive symptom (e.g., “I feel sad nearly all the time”). A total score is derived (possible range: 0–84), and higher scores are indicative of relatively severe depressive symptomatology. From the 1799 participants, 29 failed to return the questionnaire and three had too many missing answers (>6 items); these were excluded from any analysis involving the IDS. The IDS showed excellent internal reliability across all those without missing answers (n = 2150; Cronbach’s α = .90).

#### Composite International Diagnostic Interview v2.1 (CIDI; [39,40])

Depressive and anxiety disorders were determined using the semi-structured CIDI (v2.1). The CIDI is used worldwide and WHO field research has found high inter-rater reliability [41], high test-retest reliability [42], and high validity for depressive and anxiety disorders [40,43]. Diagnosis of MDD, dysthymia, panic disorder (with and without agoraphobia), generalized anxiety disorder, social anxiety and agoraphobia were determined based on the criterion outlined in the DSM-IV. MDD episode severity was determined by the number of nine possible depressive criteria met (including the two core symptoms). Number of previous MDD episodes was asked at baseline when participants indicated a history of (or current) MDD. A total number of MDD episodes was derived by adding the number of waves where an MDD episode was reported to the number of previous MDD episodes reported at baseline. Age of onset at first MDD/AD episode was also asked when participants reported an MDD or AD disorder since the previous interview. Trained research staff conducted the interview.

#### Implicit Association Test (IAT; [44])

Implicit self-esteem was measured with a self-esteem version of the computer-based IAT. The IAT is a word-sorting task where words are presented from two target categories: *I* (I, myself, self, my, own) and *other* (other, you, they, them, themselves); and two attribute categories: *positive* (meaningful, successful, important, worthwhile, confident) and *negative* (worthless, unimportant, weak, failure, useless; translated from Dutch). Following two practice rounds of ten trials, participants sorted *positive*- and *I*- related words with the same key and *negative*- and *other*- related words with the other key (pairing 1). This was repeated for two blocks of 20 trials. Participants then completed another practice block of ten trials with only attribute words, although key allocation had been swapped. Participants ended the task with two blocks of 20 trials where *negative*- and *I*- related words (and *other*- and *positive*- related words) shared the same key (pairing 2). Reaction time of the initial response and accuracy were recorded. The premise of the IAT is that the attribute and target categories that are more strongly associated for the participant are easier to sort when they share a key. A person with high implicit self-esteem is therefore expected to find it easier to sort words when *I* and *positive* share a key than when *I* and *negative* share a key.

The IAT was scored based on the D_4_-measure [45]. First, trials with reaction times longer than 10,000 ms were discarded. Reaction times on error trials were replaced with the mean of the correct answers in that block with an added 600 ms error penalty. The mean reaction time for pairing 1 was then subtracted from the mean reaction time for pairing 2, then subsequently divided by the pooled standard deviation of both pairings to control for individual variation. Higher scores were therefore indicative of a relatively fast response when categories “I “and “Positive” share a key, thus indicating higher implicit self-esteem. Participants were excluded from any analysis involving IAT scores when more than 10% of trials were faster than 300 ms, an error rate of over 20%, or where more than 1% of trials were longer than 10,000 ms (n = 114; [46,47]). Spearman-Brown corrected correlation between test halves was .85 (test halves based on trials 1, 2, 5, 6, etc., and 3, 4, 7, 8, etc.).

#### Rosenberg Self-Esteem Scale (RSES; [48])

Explicit self-esteem was measured with a self-report questionnaire containing 10 items answered on a 4-point Likert scale from 1 (*strongly agree*) to 4 (*strongly disagree*). Higher scores (possible range: 0–40) were indicative of more positive self-esteem. The measure showed good internal reliability in the present study (Cronbach’s α = .92; based on all 1799 participants).

#### Scale for Suicide Ideation (SSI; [49])

The first five items from the original 19-item SSI were included in NESDA. These items were initially used as a screening instrument to identify those who had active or passive suicidal ideation before receiving the rest of the questions to gain further insight into the severity and attitudes of the suicidal ideation. The first five questions (e.g., “What feelings did you have last week about dying. Did you want to die and how strong was this wish”) were asked in a semi-structured interview with answers given on a three point scale, where 0 indicated no suicidal ideation and 2 indicated moderate to strong suicidal ideation.

A dichotomous variable was created to identify those with suicidal ideation (1) and those without (0) in the current MDD group. Suicidal ideation was quantified by a score above zero on the SSI and on item 18 of the IDS [50]. Participants who scored 0 on both were identified as not having suicidal ideation. Those who scored a zero on one measure, and higher on the other, were excluded from the analysis.

### Procedure

NESDA assessments take between three and five hours, and are completed in one sitting [35]. Assessments contain computer tasks, self-report questionnaires, interviews, and biological measures carried out by trained staff. In all cases, participants completed the RSES after the IAT. Participants received travel expenses and a 15 euro gift certificate.

### Statistical Analysis

In the first part of the analysis, a MANOVA was conducted with RSES and IAT scores as dependent variables, and group as the independent variable (current MDD, remitted MDD, current AD, remitted AD, current CM, remitted CM, recovered MDD, recovered AD or controls). Univariate extreme outliers in RSES and IAT scores, per group, were standardized values exceeding ±3.3 and omitted when present. In order to test for multivariate outliers, Mahalanobis distance was calculated by regressing participant ID number onto RSES and IAT scores for each group. With 2 degrees of freedom, and a critical alpha of .001, the critical chi-square value was 13.82. Mahalanobis values exceeding this were excluded from the MANOVA as they were considered multivariate outliers. Homogeneity of variance-covariance matrices was checked using Box’s M. According to Tabachnick and Fidell, robustness is not guaranteed when sample sizes are unequal and Box’s M test is significant at *p* < .001. We reported Pillai’s Trace for the multivariate tests, and conducted Sidak comparisons to adjust for multiple testing. Cohen’s d, unadjusted for the multiple testing, is reported for each significant comparison to give an indication of the effect size and as such, reported p-value (adjusted for multiple testing) and CI for the Cohen’s d (not adjusted for multiple testing) may differ (e.g., the latter may contain a zero while the give p-value is significant). Comparisons between explicit self-esteem scores were conducted on means slightly different than those reported in Table 1 given that a number of participants had been excluded from the analysis based on invalid IAT scores.

In the final part of the analysis, looking at discrepant self-esteem (i.e., the extent that ISE and ESE differ), two logistic regressions were conducted: current MDD group (vs. controls), and current AD group (vs. controls). The absolute difference between standardized scores of the IAT and RSES were computed for all participants. Two discrepant self-esteem variables were created: damaged self-esteem and fragile self-esteem. The damaged self-esteem variable was computed by taking the absolute difference when IAT was higher than RSES; a 0 was assigned for participants where RSES is higher than IAT. Likewise, the fragile self-esteem variable was computed by taking the absolute difference when RSES was higher than IAT; a 0 was assigned for participants where the reverse was true. As such, for each participant, an absolute difference score appeared in either the damaged self-esteem variable or the fragile self-esteem variable, and had a score of 0 in the other discrepant self-esteem variable. The two discrepant self-esteem variables were entered at step one, with raw score on the RSES entered at step two.

## Results

### Missing Data Analysis & Descriptives

In order to check whether those who had received the self-esteem measures (i.e., completers, n = 1799) were not systematically different from those who had not (i.e., non-completers, n = 457), comparisons were made on BAI, IDS, and age. There was no evidence that the mean age of non-completers (M = 46.81, SD = 12.80) differed from completers (M = 48.05, SD = 13.18), *t*(2254) = 1.81, *p* = .07, *d* = -0.09, 95% CI [-0.20, 0.01]. Scores on the BAI and IDS were significantly positively skewed for both groups, and thus square-rooted (mean raw scores reported for interpretability). Results based on equal variances not assumed suggested that non-completers had higher BAI scores than completers (M = 8.40, SD = 9.64 versus M = 7.43, SD = 7.95), *t*(518.02) = 4.57, *p*< .001, *d* = 0.28, 95% CI [0.17, 0.39], and higher IDS scores (M = 17.95, SD = 13.85 versus M = 14.52, SD = 11.31), *t*(516.93) = 4.04, *p* < .001, *d* = 0.25, 95% CI [0.13, 0.36].

Means and standard deviations of the various outcome and predictor variables are given in the lower half of Table 1. Transformations did not correct data skew for IAT, RSES, BAI and IDS scores (Shapiro Wilk’s > .97, *p*’s < .001) in the complete sample, and therefore Spearman’s Rho is reported. There was a small but statistically significant positive correlation between ISE and ESE, ρ(1600) = .18, *p* < .001. Further, there were small but statistically significant negative associations between ISE and symptoms of anxiety, ρ(1570) = -.09, *p* = .001, and depression, ρ(1572) = -.12, *p* < .001, which disappeared once statistically controlling for ESE, ρ(1569) = .02, *p* = .55 and ρ(1571) = .01, *p* = .78, respectively. ESE was strongly correlated with symptoms of anxiety, ρ(1684) = -.55, *p* < .001, and symptoms of depression, ρ(1685) = -.67, *p* < .001. Anxiety and depression symptomatology were also highly correlated, ρ(1682) = .78, *p* < .001. This correlation decreased once controlling for ESE, r(1681) = .68, *p* < .001, and barely changed once controlling for ISE, r(1568) = .77, *p* < .001.

Within the current MDD group, 29 participants indicated no suicidal ideation and 14 did. Seventeen were excluded based on inconsistent answers on the SSI and the suicide-related item on the IDS. An independent samples t-test indicated that those with suicidal ideation reported lower ESE (M = 23.86, SD = 5.01) than those without (M = 28.17, SD = 5.13), *t*(41) = 2.60, *p* = .01, *d* = -0.85, 95% CI [-1.53, -0.16]. Excluding 5 with invalid IAT scores, there was no evidence of a difference in ISE between those with suicide ideation (M = .68, SD = .61) and those without (M = .54, SD = .40), *t*(36) = .81, *p* = 0.42, *d* = -0.29, 95% CI [-1.02, 0.44]. Those with suicidal ideation (M = 35.71, SD = 10.78) did report substantially more depressive symptoms than those without (M = 23.00, SD = 7.20), *t*(41) = 4.59, *p* < .001, d = -1.50, 95% CI [-2.23, -0.76].

To explore differences in self-esteem between types of AD, two one-way ANOVAs were conducted. Differentiating between social anxiety disorder (n = 35), panic disorder (with or without agoraphobia; n = 21), agoraphobia (n = 26), and general anxiety disorder (n = 9), participants from the current AD groups were excluded if another AD was present in the previous six months (i.e., comorbidity within AD). Results indicated that while there was no evidence of a difference in ISE, F(3, 82) = 0.05, *p* = .98, partial η^2^ = 002, there was a difference in ESE, F(3, 87) = 5.00, *p* < .01, partial η^2^ = .15. Post-hoc t-tests (applying a Bonferroni corrected α = .008) indicated that those with a social anxiety disorder (M = 26.80, SD = 4.56) had lower ESE than those with panic disorder (M = 30.90, SD = 4.29), *t* (54) = 3.34, *p* = .002, *d* = -0.92, 95% CI [-1.50, -0.34], and those with agoraphobia (M = 30.31, SD = 4.23),*t* (59) = 3.06, *p* = .003, *d* = -0.79, 95% CI [-1.33, -0.26]). There was no evidence of further differences between AD types.

### Differences in Self-Esteem between Clinical Groups and Controls

A MANOVA following the exclusion of two extreme univariate outliers revealed a Box’s M that did not exceed the critical cut-off point of .001 (*p* = .03). The multivariate test was significant suggesting an overall difference in self-esteem across groups, F(16,1726) = 30.36, *p* < .001, partial η^2^ = .22. This was true for both ISE, F(8,863) = 4.95, *p* < .001, partial η^2^ = .04, and ESE, F(8, 863) = 80.91, *p* < .001, partial η^2^ = .43. Sidak comparisons suggested that those in the control group had significantly higher ISE than those in the current CM group (*p* < .001, *d* = 0.78, 95% CI [0.50, 1.06]), but with no evidence that it was higher than those in the remitted CM group, *p* = .98, *d* = 0.48, 95% CI [-0.07, 1.04]. Those who had recovered from MDD or AD also reported higher ISE than those in the current CM group (*p*’s = .02, *d*’s = 0.54, 95% CI [0.22, 0.85] & 0.58, 95% CI [0.25, 0.92], respectively). There was no evidence that those in the control group differed in ISE from those in the current AD group, *p* = .15, *d* = 0.32, 95% CI [0.11, 0.55], nor from those in the remitted AD group, *p* = .12, *d* = 0.59, 95% CI [0.20, 0.99]. There was no evidence of further differences in ISE between the control group and the other clinical groups, nor between the clinical groups (*p*’s > .21). For ESE, the control group had significantly higher levels than all the current, remitted and recovered groups (*p*’s < .01, d’s = 0.66–2.95). Further, those who had recovered from MDD and those who had recovered from AD had higher ESE than all remitted and current groups (*p*’s < .05, d’s = 0.70–2.12) with the exception of remitted AD (*p*’s = .46 & .99, respectively). There was no evidence of a difference in ESE between those who had recovered from MDD and those who had recovered from AD, *p* = .99, *d* = 0.19, 95% CIs [-0.08, 0.46]. Further, those with a current CM had lower ESE than all MDD and AD groups (i.e., remitted & current; *p*’s < .01, d’s = 0.70–1.43), and lower than those remitted from CM (*p* = .02, d = -0.92, 95% CI [-1.55,- 0.29]). Finally, those with a current MDD reported lower ESE than those with a remitted AD (*p* = .01, d = 0.72, 95% CI [-1.20, -0.24]). There was no evidence of further differences between the clinical groups (*p*’s >.19).

### Discrepant Self-Esteem

A two-step logistic regression was conducted to see whether discrepant self-esteem (step one) would predict current MDD from controls, and whether this would remain once including ESE (step two). In the control condition, 238 participants had fragile self-esteem (e.g., standardized RSES>IAT) and 119 had damaged self-esteem (e.g., standardized IAT>RSES). In participants with a current MDD, 15 had fragile self-esteem and 40 had damaged self-esteem. Correlations between ESE and fragile self-esteem, *r*(410) = .51, *p* < .001, and ESE and damaged self-esteem, *r*(410) =.-66, *p* < .001, were high but not multicollinear. Following the exclusion of eight extreme outliers, 88.4% of the participants would have been predicted accurately based on chance alone (i.e., a model without predictors). This accuracy increased to 90.3% (Nagelkerke’s $R_{N}^{2}$ = .29) with the inclusion of absolute discrepancy for damaged self-esteem, Wald = 21.11, *p* < .001, OR = 3.03, and the absolute discrepancy for fragile self-esteem, Wald = 5.33, *p* = .02, OR = .32). With the inclusion of RSES scores, the accuracy increased to 93.1% (Nagelkerke’s $R_{N}^{2}$ = .63). There was no evidence that damaged self-esteem, Wald = .09, *p* = .77, OR = .91, or fragile self-esteem, Wald = .09, *p* = .76, OR = 1.17, contributed significantly to this model. ESE was a significant coefficient, suggesting relatively higher levels decreased the odds of current MDD, Wald = 44.51, *p* < .001, OR = .60.

A similar two-step logistic regression was conducted to differentiate current AD from controls. In participants with a current AD, 40 participants had fragile self-esteem and 66 had damaged self-esteem. Correlations between ESE and fragile self-esteem, *r*(461) = .52, *p* < .001, and ESE and damaged self-esteem, *r*(461) = -.65, *p* < .001, were high but not multicollinear. Following the exclusion of eight extreme outliers, a model without predictors had an accuracy of 78.5%. This accuracy increased to 79.8% with the inclusion of the discrepancy self-esteem variables (Nagelkerke’s $R_{N}^{2}$ = .18). Both damaged self-esteem, Wald = 18.20, *p* < .001, OR = 2.37, and fragile self-esteem, Wald = 6.69, *p* = .01, OR = .55, were significant predictors. With the inclusion of RSES scores in the model, the accuracy increased to 85.9% (Nagelkerke’s $R_{N}^{2}$ = .53). Neither damaged self-esteem, Wald = 1.51, *p* = .22, OR = .73, nor fragile self-esteem, Wald = .72, *p* = .40, OR = 1.26, showed evidence of being significant determinants in the model. RSES scores was a significant determinant, Wald = 78.26, *p* < .001, OR = .64, suggesting that a relatively high ESE considerably reduced the odds of being in the current AD group.

## Discussion

The key findings of the present study were: i) Implicit self-esteem was lower in current comorbid individuals compared to a healthy comparison group; ii) Explicit self-esteem was lower in all current, remitted, and recovered clinical groups than the healthy comparison group; iii) Explicit self-esteem in current MDD and AD was not lower than in remitted MDD and AD, respectively. However, self-esteem was lower than in recovered MDD and AD. iv) There was no support for discrepant self-esteem in current MDD and current AD, once statistically controlling for explicit self-esteem.

Previous studies have consistently found lowered ESE in individuals with MDD and AD [1,2]. It is not particularly surprising that current MDD had lower ESE in both previous studies and the present study given that low ESE is a possible symptom of MDD. Despite this, ESE was not lower in MDD than in AD, where low self-esteem is not a symptom per se. This may highlight low self-esteem as a transdiagnostic factor explaining why comorbidity between depression and anxiety is so high [24]. Some argue that depression and self-esteem have overlapping causal factors, which may explain some of the shared variance. For example, Neiss et al. [51] concluded that there was a common genetic and environmental influence on self-esteem, negative emotionality and depression, based on stronger associations in monozygotic twins, compared to dizygotic twins. In the present study, although self-esteem in current MDD and current AD was similarly low, the correlation between symptoms of depression and anxiety remained high when partialling out ESE. Although this is consistent with the findings from a previous cross-sectional study [15], it need not mean that self-esteem does not increase the risk of developing a comorbidity. A longitudinal design is required to see which aspects of a current depression or anxiety, like low self-esteem, is related to increased risk for comorbid anxiety and depression, respectively. Highlighting which symptoms to target first to prevent comorbid disorders from developing may be particularly important given that comorbid AD/MDD is more persistent and treatment-resistant than purer forms of MDD and AD [52].

Despite the indisputable presence of low ESE in AD and MDD, how it relates to anxiety and depression remains an important question. Low self-esteem has also been argued to have a causal role in the development of AD and MDD, as lower levels are often observed to precede symptomatology [4]. Through social disruptions, increased self-focus, rumination, and lack of ability to negate or minimize the effects of anxious or threatening thoughts and feelings, low self-esteem could make a person more vulnerable to the development of AD and MDD symptomatology [4,5]. This may explain why those with social anxiety disorder had lower ESE than both panic disorder and agoraphobia, given both the social-related aspects and feelings of anxiety that may arise in low self-esteem. Interventions that focus specifically on increasing self-esteem might prevent both MDD and AD from developing. For example, in competitive memory training, positive self-images are made more salient and therefore increases the likeliness that the positive self-image is activated when the self is brought to attention. This intervention was found to improve self-esteem and depressive symptomatology in addition to treatment as usual in patients with MDD [53], but it remains to be seen whether it can also be used in prevention. Given the nature of self-report measures (e.g., the awareness that answers will be seen by someone else), negative self-evaluations are purposefully self-endorsed in MDD and AD, self-esteem interventions should not only aim to change the content of self-related evaluations but also aim to learn how to oppose negative self-evaluations that arise.

ISE in relatively pure MDD and AD was found not to differ from controls. In other words, self-related negative thoughts occur when purposefully thought about, whereas negative self-associations do not already arise at the automatic and reflexive level. For MDD, this does not appear to be explained by the presence (or absence) of suicidal ideation as was found in a previous study [23]. The lack of low ISE in MDD is consistent with many findings (e.g., [20]) but conflicts with Risch and colleagues [22]. Given that the same measure of ISE was used, explaining the conflicted findings is not straightforward. As we found lower ISE in the current CM group, the presence of AD in the MDD group may account for the lower ISE observed in their study. In the previous study where low ISE was observed in individuals with social anxiety disorder (n = 33), 40% also reported a comorbid depressive disorder [16]. Although in this earlier study a comparison of those with and without a depressive disorder suggested no difference in ISE, the small sample sizes may have limited the power to detect an effect. Previously reported low ISE in AD as well as MDD might be explained by the unaccounted presence of comorbidity. Then together with the numerous previous cross-sectional studies using clinical populations where no difference in ISE was found [20], one would be tempted to conclude that low ISE is not a feature of pure AD and MDD. However, if ISE is formed following prolonged periods of ESE that becomes overlearned, as argued by many (e.g., [8]; although see [54]), the low ESE prevalent in MDD and AD should eventually manifest in lower ISE. Another assumption of this dual processing model, is that the level of ESE is stable, before it becomes overlearned and automatic [28]. However, highly unstable ESE has been argued to be a larger vulnerability factor for MDD and AD than consistently low ESE [55,56]. As such, if ESE is particularly unstable in MDD and AD, then this may also explain why the findings involving ISE are relatively inconsistent.

The role of prolonged low ESE in the development of low ISE may explain why low ISE was observed in comorbid AD and MDD. There is some indication that the duration of symptomatology in CM would be longer given that remission rates for CM between baseline and two-year follow-up in NESDA were a lot lower, and more months with symptomatology were reported, than those with either MDD or AD alone. This is despite those with CM were more likely to seek treatment.[52]. Prolonged periods of MDD and AD might give rise to longer periods of (stable) low ESE, and this in turn might explain why lower ISE was observed in CM only. Future studies looking at the duration, severity, and stability of low self-esteem, should look at whether the duration of symptoms in current clinical groups is associated with low ISE. Alternatively, more frequent measures of ESE over a period of time might be a more precise way of looking at the role of ESE in ISE, and could also be used to look at the role of self-esteem stability in clinical disorders [57]. Untargeted negative self-evaluations at the implicit level may also explain why those with CM are more likely to show a poorer response to treatment (e.g., [58]). Concerning self-esteem scars in those with a previous MDD and/or AD, ISE was not lower in remitted and recovered AD/MDD in comparison to controls. However, this is perhaps unsurprising given that current AD and MDD also did not display lower ISE. ESE in participants with a previous MDD or AD was significantly lower than those who had never had a MDD or AD. This might partially explain the highly recurrent nature of both disorders given that low ESE is argued to increase the vulnerability for symptomatology [4], and may suggest the need for a self-esteem intervention in those who have recently recovered or remitted from AD or MDD. Interventions for remitted MDD, in general, have been shown to be effective in reducing relapse risk [59].

Damaged self-esteem increased, while fragile self-esteem decreased, both the likelihood of current MDD and current AD, compared to controls. This is consistent with previous findings [31], although we extend this by highlighting that discrepant self-esteem variables were no longer significant once controlling for the main effect of ESE. Previous methodology either did not allow for the inclusion of ESE in the model [31,33], or did not allow for the specification of the direction of discrepancy (i.e., using the interaction between ISE and ESE to represent discrepancy). Although the current methodology allows for the inclusion of one main effect, it does not allow the direction of the discrepancy to be taken into account (i.e., damaged vs. fragile) regardless of the extent of discrepancy which has been argued to be an important factor in discrepant self-esteem [33]. However, the relevance of such a variable is debatable. Even if one were to select only those with discrepant self-esteem (i.e., excluding those with fairly congruent self-esteem), it is theoretically still anticipated that a relatively large discrepancy in one specific direction would have a stronger association with symptomatology compared to a smaller discrepancy within the same direction. Therefore, the method used in the present study not only allows for the inclusion of one main effect, but also eradicates variables that are difficult to interpret.

Although we feel that the method used in the current study is an improvement, there are still a number of limitations. In using the IAT and RSES to derive at measures of discrepancy there is an assumption that the two measures only differ on the construct they tap into. However, the RSES refers to explicit self-esteem in the past seven days, while in the IAT self-esteem is measured at that specific moment. Further, although ISE is anticipated to be a trait rather than a state, it is also argued to be context-dependent. As such, the lab settings may have an influence. For ESE, participants are able to reflect to more natural, daily situations. As such, not just a difference in timing, but also a difference in context might be measured. Ideally, a measure would be developed that directly taps into self-esteem discrepancy. We do not reject the notion of discrepant self-esteem, necessarily, but current methods (present study including) of combining ISE and ESE measures may introduce too much noise for quantifying real discrepancies.

### Limitations

Little can be said about the causal relationship of self-esteem in MDD and AD given the correlational and cross-sectional design of the present study. As low ESE was prominent across most clinical groups, the next logical step would be to study whether manipulating self-esteem influences symptomatology. There is some support that this may be the case given that self-esteem interventions also showed beneficial effects on symptoms of depression and anxiety [53]. However, it is also theoretically feasible that low self-esteem is the by-product of symptoms. Experience sampling might be a more elegant way of looking at whether self-esteem precedes symptoms or symptoms precedes self-esteem, particularly as many studies argue that self-esteem fluctuates on a daily basis in response to negative and positive events [60].

Even within the large scale NESDA study, both remitted AD (n = 29) and remitted comorbidity (n = 14) had comparatively small groups, and therefore the possibility to detect an effect may have been limited. Small clinical samples are relatively common in research [19,22] because, for example, recruitment can be difficult (especially if one needs relatively “pure” groups), and drop-out rates are high. The power to detect a difference between remitted comorbidity (i.e., the smallest group) and the healthy comparison group was 83% for a large effect (.80), 45% for a medium effect (.50) and 11% for a small effect (.20) [61]. As such, there was limited power to detect medium and small effects, which may have resulted in type II errors. However, the limited sample size is unlikely to have inflated the Type I error. Future studies need to focus on recruiting larger samples of remitted AD and remitted comorbidity, as these, particularly, were underpowered in the present study.

There are a number of critics of the IAT, and other measures of ISE. Many criticisms concern the lack of applicability with regards to using cut-off scores (e.g., above a specific score is indicative of an implicit racial bias; [62]. However, even when comparing scores on the IAT between groups, as in the present study, a number of criticisms remain relevant. One of the more recent criticisms concerns the inability for the IAT to highlight how attributes and targets are related (e.g., differentiating between implicit ideal self and actual self [63]). Despite promising findings in differentiating between ideal self and actual self in dysphoric students [63], research is needed to justify the notion of implicit goal-oriented constructs (i.e., construct validity of implicit ideal self). Also given the relatively more obvious nature in the way it was measured (i.e., “I am” and “I want to be” remained onscreen during the word-sorting task), it is unclear in how far this may have triggered explicit processing thereby influencing the speed with which words were sorted. Further criticism comes from the low validity of ISE measures, which have led some to doubt whether ISE actually exists. One common argument stems from the validity of ISE measures in comparison to the validity of ESE measures (e.g., [64,65]). Validity of ESE measures is likely to be overinflated given that biases and measurement error is likely to apply to all measures of ESE (i.e., inflated convergent validity), and is likely to apply to self-report measures of other constructs (i.e., inflated predictive validity). For example, self-enhancement bias is not only, presumably, going to affect all self-report measures of ESE, but also self-report measures of depressive symptomatology. As such, it is unsurprising that self-report measures of ESE often trump implicit measures of ISE. Another line of criticism regarding IAT and ISE seems to stem from the conceptual misunderstanding that a single self-esteem exists which can be accessed implicitly or explicitly. Doubts are then voiced because of the lack of correlation between ISE and ESE measures which is often lower than other implicit and explicit constructs [66]. However, theories postulate that ISE is best considered as the most primitive self-evaluation. With increasing time, cognitive resources and motivation, other processes are activated that may alter, overrule, or support the initial reaction. Indeed, when encouraged to rely on their gut-feeling (i.e., intuition), correlations between ESE and ISE increase [67]. There are many possible reasons as to why ESE does not correlate with ISE (e.g., self-enhancement bias, self-protection bias, narcissism), and not all apply to other implicit/explicit constructs. This is why correlations between the two are often reported to be very small or non-existent, and may explain why correlations in other implicit/explicit constructs are higher [66]. ISE is likely to be considered to contain more truth value in a person who values their “intuition” or “gut feeling” highly, or does not have the cognitive resources to carry out ESE elaborately, in which ISE evaluations may carry more weight. Undeniably, the IAT is no perfect measure of ISE, and our understanding of ESE and ISE certainly requires further refinement. However, previous research has demonstrated the IAT to have high validity [10,11,68] and reliability [12,45] and as such, completely discarding the IAT as a measure of ISE is not entirely justified.

## Conclusions

The present study was the first to compare levels of both ESE and ISE across various phases of MDD and AD groups and with careful consideration of comorbidity. We found that consistent across all clinical groups, at all phases, a more negative, self-evaluation was endorsed in comparison to the control group. In other words, conscious behaviour consistent with low ESE is more likely to manifest in MDD and AD, and remain during remittance and recovery. However, negative self-evaluations at the implicit level were only evident in individuals with current CM, while levels of ISE in relatively pure forms of MDD and AD were equivalent to the controls. This may not only highlight why CM is more persistent and treatment-resistant, but also emphasizes the need for future research to investigate whether the aetiology of CM differs from MDD or AD. As such, it is also important to control for the potential presence of a comorbid disorder when further examining the relevance of ISE (and ESE) in AD or MDD.
